# Extensive spinal epidural abscess caused by Staphylococcus epidermidis: A case report and literature review

**DOI:** 10.3389/fsurg.2023.1114729

**Published:** 2023-03-08

**Authors:** Yang-wei Pi, Yan Gong, Jia-jia Jiang, De-jin Zhu, Yue-xin Tong, Li-ming Jiang, Dong-xu Zhao

**Affiliations:** Department of Orthopaedics, China-Japan Union Hospital of Jilin University, Changchun, China

**Keywords:** spinal epidural abscess (SEA), staphylococcus epidermidis, laminectomy, drainage, antibiotic therapy

## Abstract

**Background:**

Extensive spinal epidural abscess (SEA) is an exceptional and threatening condition that requires prompt recognition and proper management to avoid potentially disastrous complications. We aimed to find key elements of early diagnosis and rational treatments for extensive SEA.

**Case presentation:**

A 70-year-old man complained of intense pain in the cervical-thoracic-lumbar spine that radiated to the lower extremity. Laboratory test results revealed a marked increase in all indicators of infection. The spinal magnetic resonance imaging (MRI) revealed a ventral SEA extending from C2 to L4. Owing to the patient's critical condition, laminectomy, drainage, and systemic antibiotic therapy were administered. And the multidrug-resistant *Staphylococcus epidermidis* was detected in the purulent material from this abscess.

**Results:**

Postoperative MRI revealed diminished epidural abscess, and the clinical symptoms were dramatically and gradually relieved after two rounds of surgery and systemic antibiotic therapy involving the combination of ceftriaxone, linezolid, and rifampicin.

**Conclusions:**

A comprehensive emergency assessment based on neck or back pain, neurological dysfunctions, signs of systemic infection, and MRI are important for early diagnosis of extensive SEA. Further, the combination of laminectomy, drainage, and systemic antibiotic therapy may be a rational treatment choice for patients with SEA, especially for extensive abscess or progressive neurological dysfunction.

## 1. Introduction

Spinal epidural abscess (SEA), i.e., an accumulation of purulent contents in the epidural space of the spinal canal, can lead to spinal medullary ischemia upon compression, resulting in progressive neurological dysfunction, systemic inflammation, and even death (1–4). A recent report indicated that SEA-associated hospitalization has increased to nearly 1/1,000 (5, 6). This trend may be due to the growing population of immunocompromised patients with cancer and diabetes, and the increasing number of invasive spinal surgeries. The mean age of SEA is 50 years with most cases occurring between the ages of 30 and 70 years, though children may be rarely affected (1, 2, 7, 8). Although typical SEA symptoms include fever, back pain, and progressive neurological dysfunction (9), approximately 50% of patients have either back pain or other non-specific manifestations (10, 11), leading to a high rate of misdiagnosis and inevitably missed diagnosis. In addition to diagnosis, the therapy and prognosis of SEA are important and can directly determine the survival rate of patients. A neurosurgeon, Dandy (12) first used laminectomy and performed direct drainage of the abscess for SEA surgery. Moreover, the emergence of antibiotics effectively reduced mortality. Therefore, a combination of these two is the rational treatment for symptomatic SEA, especially for extensive spinal cord compression and progressive neurological dysfunction (13, 14). However, the prognosis of SEA remains not optimistic, with 4%–22% of patients having severe neurologic disability such as irreversible paraplegia or other deficits occurring, and less than half of patients return to baseline neurologic status (15–19). Mortality rates range from 2% to 20% (1, 2, 15, 16, 19, 20).

According to Reihsaus et al. (10), only 1% patients presented with extensive SEA spanning the cervical-thoracic-lumbar region. For the treatment of extensive SEA, the majority of neurosurgeons and spine surgeons favor aggressive surgical approaches. The techniques include single- or multi-segment laminectomy, followed by irrigation or sufficient epidural drainage to remove purulent secretions (4, 21, 22). Determining the scope of surgery and minimizing surgical trauma with proper drainage are crucial for extensive SEA treatment.

In August 2022, a case of extensive SEA spanning the cervical-thoracic-lumbar region caused by *Staphylococcus epidermidis* was successfully treated at our department. Herein, we analyze and discuss the diagnosis and treatment experience.

## 2. Case presentation

A 70-year-old man was admitted to the hospital with a visual analog scale (VAS) score of 7 and a body temperature of 38.5°C due to acute discomfort in the cervical-thoracic-lumbar spine that radiated to the lower extremity. He had back and neck pain for two weeks, which restricted the spine's range of motion, affected his lower limbs, and caused physical weakness. He reported a history of diabetes mellitus and underwent lumbar discectomy and transpedicular screw fixation (L4/5) 4 months previously.

On admission, the muscle power was 3/5 in the left lower extremity but normal in the other extremities. Reflexes were reduced in the quadriceps and ankle jerks. The left straight leg raise test result was positive at 30°. Negative pathological reflexes were observed in the upper and lower extremities along with neck stiffness and positive Brudzinski signs.

**Figure 1:**
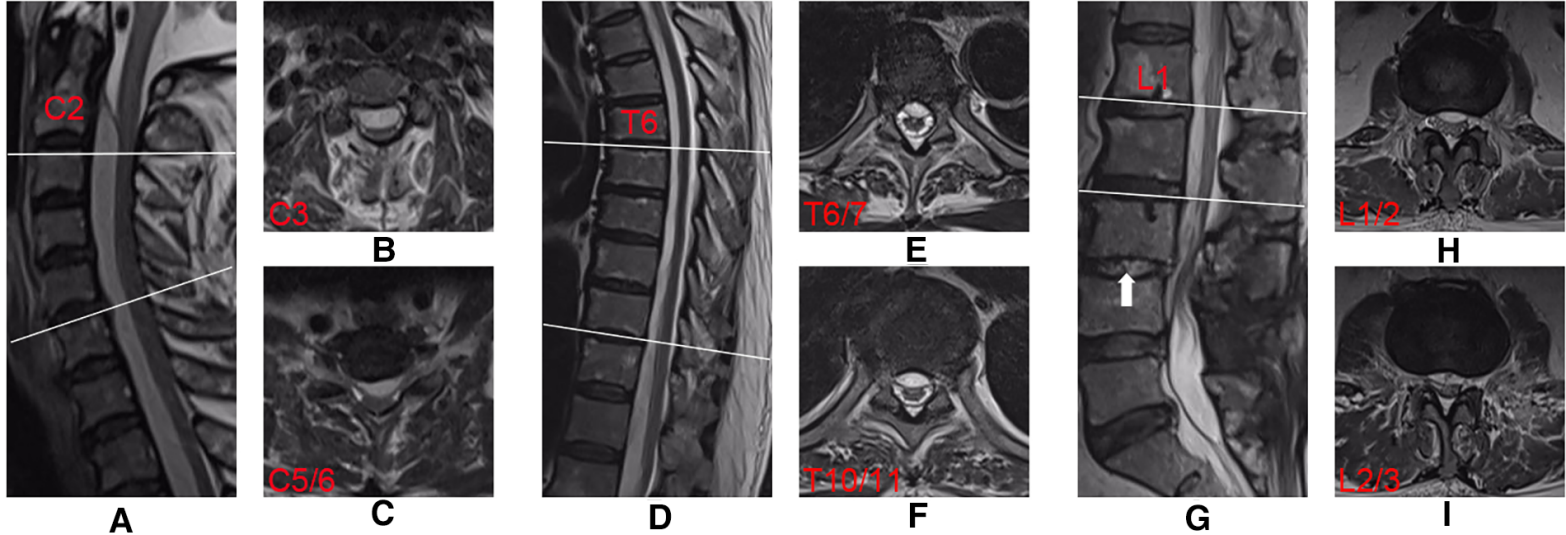
Preoperative T2-weighted MRI sequences obtained from an extensive SEA in a 70-year-old man. Midsagittal views showing the SEA (**A,D,G**). Axial imaging at C-3 (**B**), C-5/6 (**C**), T-6/7 (**E**), T-10/11 (**F**), L-1/2 (**H**) and L-2/3 (**I**) showing extensive ventral collection. Mixed-high signals (white arrow) on T2-weighted imaging indicate the spondylodiscitis at the L3/4 segment (**G**).

Laboratory test results showed a marked increase in white blood cell (WBC; 13.99 × 10^9^/l), erythrocyte sedimentation rate (ESR; 78.00 mm/h), and C-reactive protein (CRP; 123.96 mg/l) levels, suggesting a severe infection. Blood culture results were negative. Magnetic resonance imaging (MRI) confirmed extensive SEA from C2 to L4 and a spondylodiscitis at the L3/4 segment (Figure 1).

**Figure 2:**
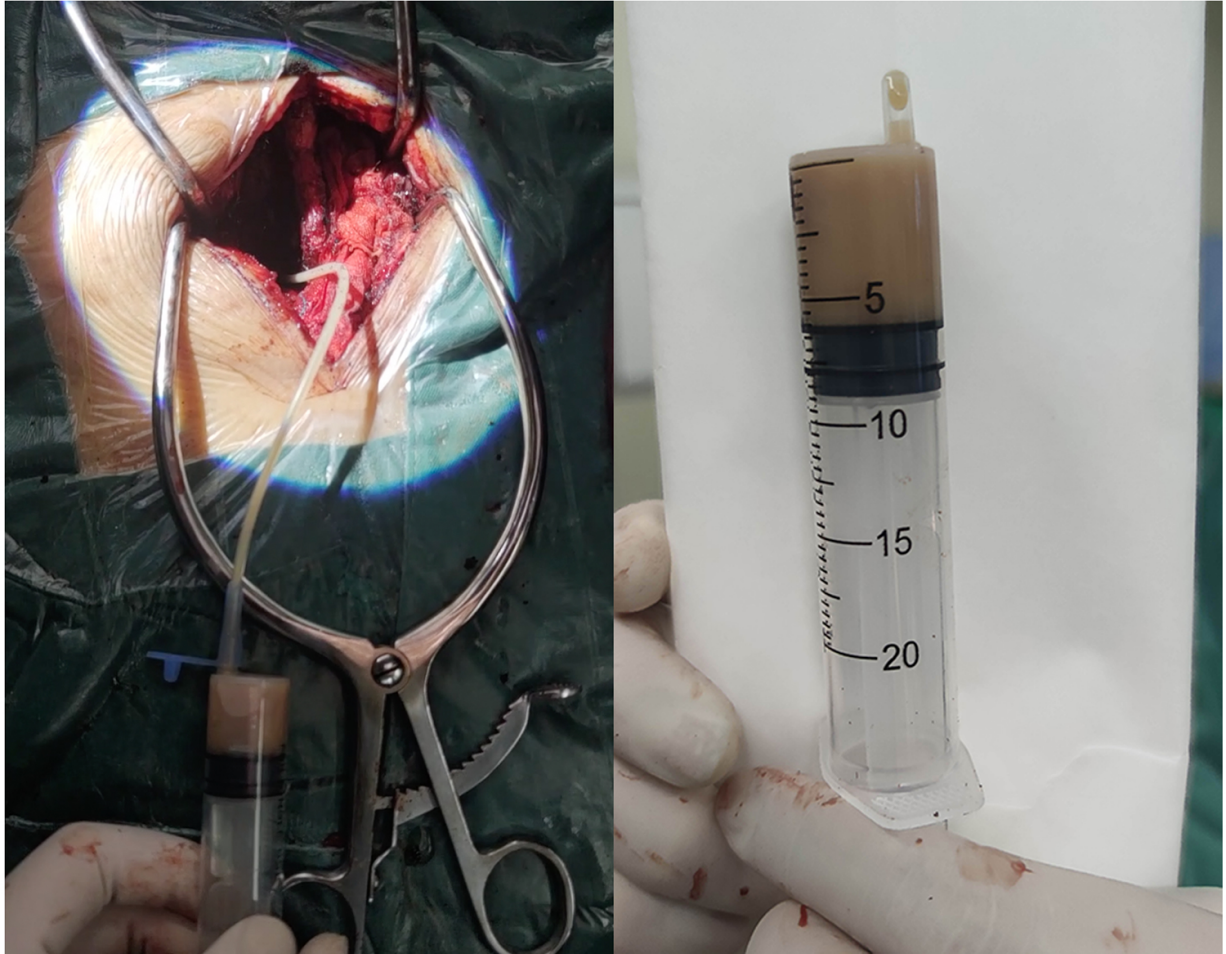
Intraoperative photographs: pale-yellow purulent fluid was drained from lumber spine.

**Figure 3:**
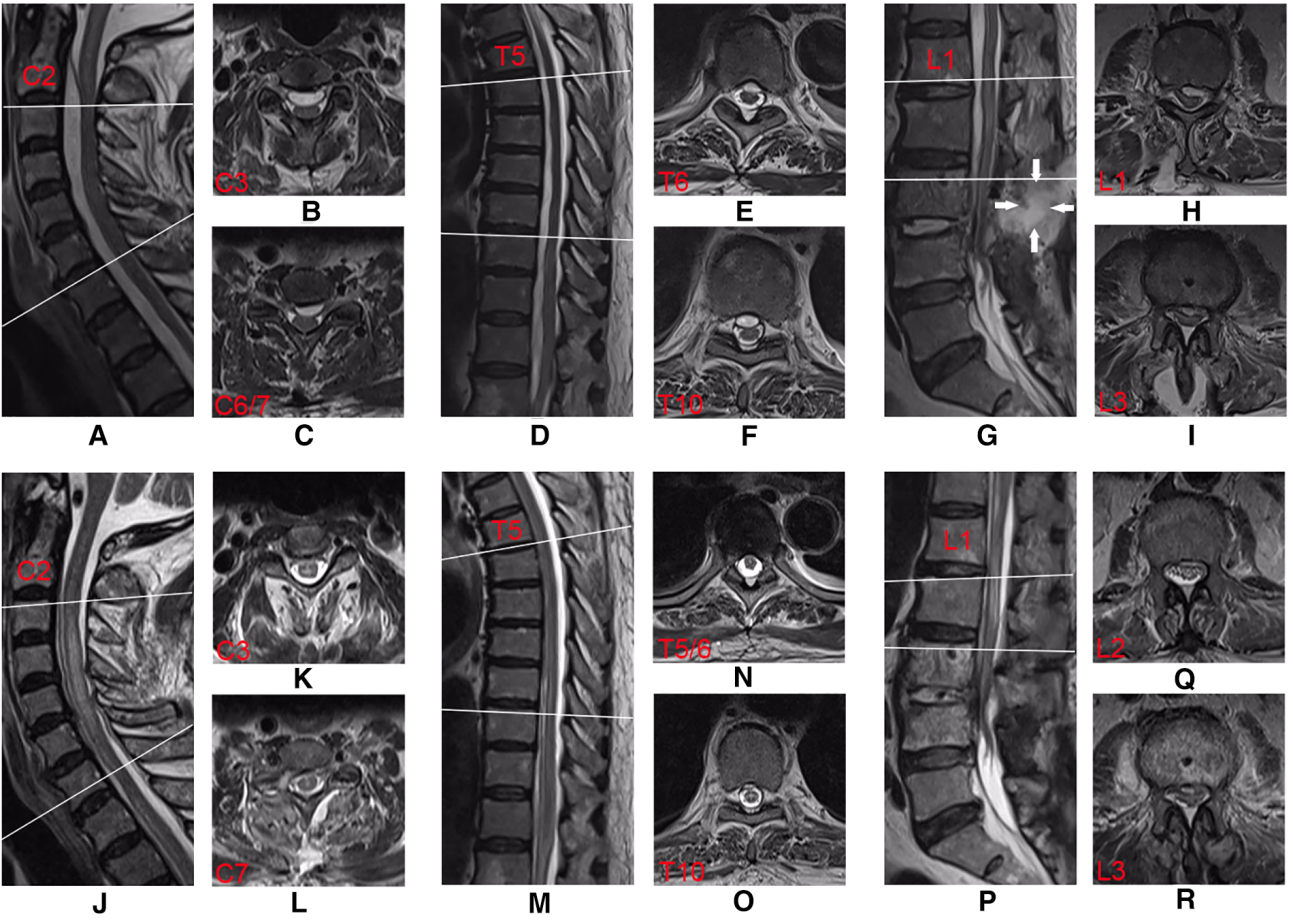
Postoperative T2-weighted MRI sequences of the first surgery (A–I) and second surgery (J–R). Comparing with pre-operation, the midsagittal view (**A**,**D,G**) illustrated slightly reduction of epidural abscess at cervical, thoracic, and lumbar levels. Mixed-high signals (white arrows) on T2-weighted imaging indicate that the lumbar incision infection was aggravated (**G**). The midsagittal view (**J,M,P**) and axial view (**K,L,N,O,Q,R**) showed the absence of epidural abscess, and the compression has significantly relieved after the second surgery.

The patient's general condition was critical, with progressive weakness of the left lower extremity. The extensive SEA was the ultimate cause of this weakness. Therefore, laminectomy and abscess drainage were performed urgently. During the surgery, the internal fixations in L4 and L5 were taken out firstly. Then, L3/4 left hemilaminectomy was performed to break through the posterior longitudinal ligament, and a pale-yellow purulent fluid was observed. A 3-mm-diameter ventricular drainage tube was gently and gradually guided cephalad along the ventral side, and the operating table was tilted (reverse Trendelenburg) for drainage (Figure 2). In total, about 20 ml of purulent fluid was drained. The collected samples were sent for Gram staining and bacteriological culturing. Irrigation and evacuation procedures were repeated until no abscess or purulent material was observed. The incision was repeatedly and carefully douched with iodine and saline with vancomycin. Finally, the incisions were closed after the negative pressure drainage vessel was retained. As the patient was allergic to vancomycin, empirical intravenous antibiotics with ceftriaxone (2.0 g/day) were administered on the right postoperative day. After surgery, the patient was placed on the bed with his head elevated and his foot lowered and drainage was continued. The patient's blood and drained pus cultures were positive for multidrug-resistant *S. epidermidis*. Antibiotic therapy was, therefore, changed to linezolid (0.6 g/day) and rifampicin (0.6 g/day) based on the antibiotic sensitivity profile of the bacteria. Seven days after surgery, the patient's lower back pain persisted, with a VAS score of 6, and the muscle power was 4/5 in the left lower extremity. Twelve days after surgery, re-examination of the spinal MRI showed that the abscess volume was slightly reduced and the pressure in the purulent cavity was relieved. However, mixed-high signals appeared in the lumbar incision on T2-weighted imaging, indicating aggravation of local infection (Figures 3A–I).

To further relieve spinal cord compression and clean up local infection and necrotic tissue of the original lumbar incision, hemilaminectomy was performed again at the center of C6/7 and L3/4 on the 14th day after the first surgery. During surgery, part of the C6/7 lamina was removed, the posterior longitudinal ligament was broken through, a ventricular drainage tube was inserted into the caudal sides along the ventral side, and about 3 ml of purulent fluid was drained. The abscess was repeatedly irrigated with saline. The incision was repeatedly and carefully douched with iodine solution and saline with vancomycin, and a negative-pressure drainage vessel was retained outside the epidural space. Thereafter, the original lumbar incision was opened again, and the local necrotic tissue was cleaned. L3/4 right hemilaminectomy was performed, and a ventricular drainage tube was guided cephalad along the ventral side. Approximately 6 ml of purulent fluid was drained, and the purulent cavity was repeatedly irrigated with saline. The intervertebral infection and lesion tissues were cleaned again; gentamicin sulfate (4 ml) and vancomycin-loaded calcium sulfate (10 ml) were added to sterile water (6 ml); the mixture was converted into artificial bone granules, approximately 3 mm in size, and implanted into the L3/4 intervertebral space. Finally, the incisions were closed after the negative pressure drainage vessel was retained.

**Figure 4:**
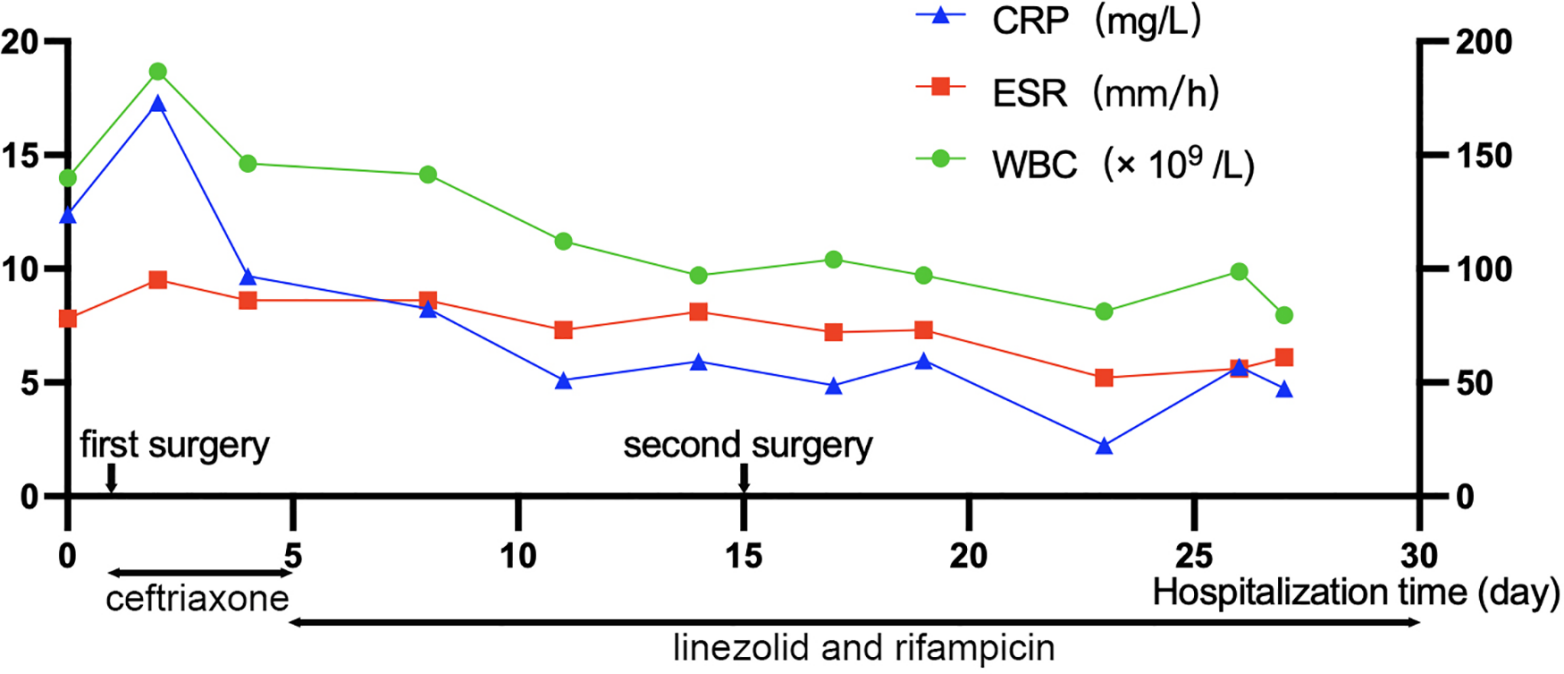
Schema of antibiotics application and time course for C-reactive protein (CRP), white blood cell (WBC), and erythrocyte sedimentation rate (ESR). The horizontal axis indicates the days of hospitalization, and the vertical axis indicates the levels of CRP, WBC, and ESR. The two-way arrows below the graph indicate the intravenous antibiotic treatment.

After surgery, linezolid (0.6 g/day) and rifampicin (0.6 g/day) were continued for anti-infection therapy. Blood and drained pus cultures were negative three days after the second surgery. Twelve days after the second surgery, pain was relieved, with a VAS score of 2, and the muscle power remained 4/5 in the left lower extremity. Re-examination of the spinal MRI showed that the abscess in the spinal canal had almost disappeared, and the compression was significantly relieved (Figures 3J–R). The schema of antibiotics application and time course for CRP, WBC, and ESR are shown in Figure 4. Thirty days after the second surgery, almost all neurological symptoms disappeared and the muscle power of the extremities was 4–5/5.

## 3. Discussion

SEA can be secondary to bacteremia caused by any factor (15), of which diabetes is a common complication, occurring in 21%–43% of patients with SEA (8). The placement of an epidural catheter is also an important risk factor for SEA, with an incidence rate of 0.5%–3.0% (23, 24). Other potential risk factors include intravenous drug injection, HIV infection, trauma, tattooing, acupuncture, and adjacent bone or soft tissue infection. Herein, the patient had a history of diabetes mellitus and lumbar internal fixation surgery, which was followed by insertion of an epidural drainage tube. We believe that this patient had a delayed infection after surgery, which caused extensive SEA, and diabetes mellitus was one of the aggravating factors.

Over 70% of SEA instances, according to Reihsaus et al. (10), are caused by systemic or localized inflammatory disorders or immunodeficiency diseases, and about 22% occur postoperatively. Methicillin-susceptible Staphylococcus aureus (MSSA) and methicillin-resistant Staphylococcus aureus (MRSA) are responsible for about 70% of infections. Escherichia coli, Pseudomonas aeruginosa, *S. epidermidis*, and other pathogens have also been discovered (25). *S. epidermidis* infection is extremely rare (26, 27). In recent years, *S. epidermidis* gradually evolved into an important opportunistic pathogen, mainly causing medical device-related infections (28, 29). It is the primary pathogen behind catheter-related infections, early newborn sepsis, joint prosthesis infections, prosthetic valve endocarditis, and other infections linked to biomedical devices (30–33). A more serious problem with medical device-related infections is that they are often caused by multidrug-resistant bacteria, which produce adhesion factors and capsules on the surface of implanted devices that reduce the efficacy of antibacterial treatment and may even require removal of implants to completely eliminate the infection (28).

Early symptoms of SEA, including fever and fatigue, are usually non-specific. The classic triad of diagnostic significance is fever, back pain, and neurological dysfunction (9). Unfortunately, <8% patients exhibit these three manifestations simultaneously (8). For severe localized back pain and fever, SEA or spinal osteomyelitis should be considered. However, when patients seek medical advice for back pain only, they are rarely considered to have SEA. Nearly 75% of patients ultimately diagnosed with SEA are believed to have diagnostic delays, several trips to the emergency room, hospitalizations without a diagnosis, or time-lag >24 h before a conclusive diagnostic test (9, 34). We believe that patients with back pain who may be suspected with SEA should undergo a comprehensive physical examination; ESR, CRP levels, and blood culture should be checked immediately. Blood culture results are often consistent with subsequent pus culture results; thus, pathogens can be determined in advance, and these results can guide early intervention and treatment. If ESR is significantly elevated, MRI should be performed immediately. Currently, MRI is a powerful tool for diagnosing SEA (35). Through standard and enhanced scanning, we can understand the condition of the vertebral body and surrounding soft tissues of the spine, the scope and composition of the abscess, the compression of the dural sac, and the degree of spinal cord injury. Moreover, through re-examination, MRI can be used as an indicator to evaluate efficacy. Additionally, noninvasive MRI can avoid the risk of infection of subarachnoid space through injection of contrast agent or puncture and aspiration; for patients with complete spinal canal obstruction, it can also prevent the aggravation of nerve injury by injection of contrast agent.

Currently, there are no randomized controlled trials comparing the results of conservative treatment (non-surgical treatment) and surgical treatment for SEA. Most reports are retrospective, single-center case studies, or case reports. Many retrospective studies have selection bias, i.e., patients in the conservative treatment group have smaller abscesses and better initial neurological functional statuses than those in the surgical treatment group (36). There is no clear consensus on the treatment of SEA, and disagreement persists between non-surgical and surgical treatments. Early literatures suggest surgical decompression for all patients with SEA, while a recent systematic retrospective study shows that an increasing number of patients with only back pain but no neurological symptoms received non-surgical treatment; there was no statistically significant difference between the results of the surgical treatment group and those of the non-surgical treatment group (25). However, for neurological symptoms or extensive compression in patients with SEA, early intervention is required to avoid irreversible neurological damage. SEA treatment completely eliminates pathogenic microorganisms and suppress purulent secretions. Early surgical decompression and drainage improve the final prognosis (37). Surgeons prefer laminectomy and decompression; however, the scope of laminectomy remains uncertain. Schultz et al. (38) suggested selective laminectomies at the rostral and caudal poles of the abscess with subsequent drainage, whereas others performed laminectomies focused at the apices of natural spinal lordosis and kyphosis of the cervical spine at C-4, C-5, or C-6, in the thoracic spine at T-6, T-7, or T-8, and in the lumbar spine at L-3 or L-4 (21). There is also a report on the surgical treatment of extensive SEA with whole spinal laminectomies (39). However, extensive multi-segment laminectomies may not be performed for the following reasons: severe or critical patient condition, risk of mechanical instability, long times of surgery, and high operative blood loss (40–42).

We reviewed the extensive SEA cases from 2010 to 2022. The electronic database PubMed was searched, and the search terms included “extensive spinal epidural abscess” or “holocord spinal epidural abscess”. 21 cases (Table 1), which the abscess covered three or more spinal regions, were ultimately selected with a wide range age, including infant (8-month-old) and older patient (81-year-old). Notably, 10 of the 21 patients had diabetes mellitus, and five patients had soft tissue infection in other parts. In 14 of the 21 patients, the abscess was dorsal to the dural sac, in (only) two, it was ventral; and in the remaining five, it was peripheral to the dural sac; 12 cases were infected by *S. aureus* (including MSSA and MRSA), and three were infected by Streptococcus. In total, 19 patients chose laminectomy and epidural catheter irrigation, but internal fixators were not used to stabilize their spines. Only two patients chose abscess puncture irrigation and drainage exclusively. Ultimately, most patients' extremity power significantly improved. Herein, most patients underwent multi-segment laminectomies and epidural catheter irrigation and drainage. Catheter irrigation reduced severity of the infection, stopped the spread of the inflammation, and successfully released spinal cord compression (22). In all cases of laminectomies, surgery at the cervicothoracic, thoracolumbar, or lumbosacral connections is never performed during laminectomies to minimize instability (6, 60, 61).

**Table 1:** Literature review of 21 extensive SEAs reported between 2010 and 2022.

Authors & Year	Patient Age & sex	Comorbidities	Preoperative power	MRI Findings	Pathogen	Surgery Performed	Epidural Irrigation Used	Instrumentation Used	Antibacterial drugs	Postoperative power
Tracz et al. (43), 2022	74, male	DM	NM	C2-S1, circumferential	Cutibacterium acnes	C3–4, T2–3, T12, L3, laminectomies	yes	no	vancomycin	NM
Lee et al. (44), 2022	58, male	DM, HBV, HD, ARI	5/5 bilat UEs, 2/5 bilat LEs	C1-L5, circumferential	*Escherichia coli*	T9-L5, ULBD	yes	no	ceftriaxone, ciproﬂoxacin	4/5 bilat UEs, 4/5 bilat LEs
Koyama et al. (45), 2021	73, male	DM, HD	2–3/5 bilat UEs, 2–3/5 bilat LEs	C1-S2, dorsal	Streptococcus	C4–5 open-door laminoplasty, T4–7 laminotomies	yes	no	Meropenem, vancomycin, ampicillin, ceftriaxone	5/5 bilat UEs, 5/5 bilat LEs
Eroshkin et al. (46), 2021	58, male	DM	4/5 bilat UEs, 3–4/5 bilat LEs	C2-L1, dorsal	MRSA	L1 laminoplasty	yes	no	Ceftriaxone, vancomycin, Moxifloxacin	5/5 bilat UEs, 5/5 bilat LEs
Roberti (47), 2020	NM, male	NM	NM	C3-S1, dorsal	MSSA	C4, T8, L4 laminotomies	yes	no	NM	NM
Fujii et al. (48), 2020	81, male	DM, COPD et al.	NM	T6-L3, dorsal	*streptococcus*	T6–7, T11–12, L2–3 puncture	yes	no	meropenem, clindamycin, vancomycin, cefazolin	NM
Supreeth et al. (49), 2019	59, female	DM, HD	3–4/5 bilat UEs, 3–4/5 bilat LEs	C1-L5, ventral	*Streptococcus pneumoniae*	L4–5 hemi-laminectomy	yes	no	Ceftriaxone, vancomycin, cefuroxime	5/5 bilat UEs, 5/5 bilat LEs
Saito et al. (50), 2019	60, female	DM, RA	NM	the intracranial space to L5, ventral	MSSA	L4–5 puncture	no	no	vancomycin, ceftriaxone, linezolid, rifampin	NM
Thomson (51), 2018	66, female	atrial flutter	4/5 bilat UEs, 4/5 bilat LEs	C1-L4, dorsal	Staphylococcus aureus	C3–4, T7–8, L1–2 laminotomies	yes	no	vancomycin, clindamycin, flucloxacillin	5/5 bilat UEs, 5/5 bilat LEs
Dang et al. (52), 2018	55, female	DM, HD, HLP	Extremity strength is diminished	C1-L4, dorsal	*Klebsiella pneumoniae*	C, T and T decompression with evacuation	yes	no	Meropenem	NM
Kurudza et al. (53), 2018	8-month, male	none	acutely paraplegic	C2-S1, circumferential	MRSA	C3, L4 laminotomies	yes	no	Bacitracin, vancomycin	NM
Okada et al. (54), 2017	65, male	bacterial meningitis	5/5 bilat UEs, 5/5 bilat LEs	C6-S1, dorsal	MSSA	T2–3, T10–11, L4–5 laminotomies	yes	no	Vancomycin, Meropenem, ceftriaxone	5/5 bilat UEs, 5/5 bilat LEs
SInatra et al. (55), 2017	15, male	Lemierre syndrome	1–2/5 bilat LEs	T1-L4, dorsal	F. necrophorum	T2-L3 hemi-laminectomy	yes	no	Meropenem	5/5 bilat LEs
Xiang et al. (56), 2016	65, female	DM	4/5 bilat UEs, 3–4/5 bilat LEs	C1-S2, circumferential	MRSA	L3–5 laminotomies	yes	no	Vancomycin, linezolid	5/5 bilat UEs, 5/5 bilat LEs
Gerald et al. (57), 2016	19, male	piriformis pyomyositis	1/5 bilat UEs, 0/5 bilat LEs	C2-S2, dorsal	MSSA	C3–7, T11, L2–4 laminotomies	yes	no	nafcillin	4–5/5 bilat UEs, 5/5 bilat LEs
Abd-El-Barr et al. (21), 2015	51, male	DM, bacteremia	3–4/5 bilat UEs, 4–5/5 bilat LEs	C2-S2, dorsal	MSSA	C4–6, T6–8, L3–4 laminotomies	yes	no	NM	5/5 bilat UEs, 5/5 bilat LEs
Abd-El-Barr et al. (21), 2015	46, male	none	4/5 bilat UEs, 4/5 bilat LEs	C1-L4, dorsal	NM	C4–6, T6–8, L3–4 laminotomies	yes	no	NM	5/5 bilat UEs, 5/5 bilat LEs
Smith et al. (22), 2014	5, male	hip & tibial abscesses	4/5 bilat UEs, 0/5 bilat LEs	C1-S1, dorsal	MRSA	C6–7, T-8, L-1 laminotomies	yes	no	linezolid	4/5 bilat UEs, 0/5 bilat LEs
Smith et al. (22), 2014	51, male	none	0/5 bilat UEs, 1/5 bilat LEs,	C1-S1 circumferential	MRSA	C1–2, L-3 laminectomies	yes	no	Linezolid, rifampin	5/5 bilat UEs, 4–5/5 bilat LEs
Verma et al. (58), 2014	35, male	none	1–2/5 bilat UEs, 0/5 bilat LEs	C4-L5, dorsal	*staphylococci*	C7, T8, laminotomies	yes	no	NM	3/5 bilat UEs, 3/5 bilat LEs
Smith et al. (59), 2010	25, male	Crohn's disease	3/5 bilat UEs, 0/5 bilat LEs	C2-S1, dorsal	NM	C5, T8, L3 laminotomies	yes	no	NM	3–4/5 bilat UEs, 0/5 bilat LEs

DM, diabetes mellitus; HBV, chronic hepatitis B virus infection; HD, hypertension disease; ARI, acute renal insufficiency; HLP, hyperlipidemia; RA, rheumatoid arthritis; COPD, chronic obstructive pulmonary disease; MRSA, methicillin-resistant *Staphylococcus aureus*; MSSA, methicillin-susceptible *Staphylococcus aureus*; NM, not mention; UE, upper extremity; LE, lower extremity; ULBD, unilateral laminotomy for bilateral decompression; C, cervical; T, thoracic; L, lumbar; S, sacral.

Eroshkin et al. (46) reported two cases of extensive SEA and successfully inserted a drainage tube throughout the spinal canal. The abscess was then fully drained and irrigated. However, in our case, it was very difficult to insert the drainage tube during surgery, and the drainage was insufficient. The main considerations were: (1) possible separations in the abscess, which hindered insertion of the drainage tube; (2) negative pressure in the abscess made suction and drainage difficult; and (3) the abscess, being a non-Newtonian fluid was highly viscous, impeding suction and drainage. During surgery, samples for bacterial culture and drug sensitivity tests indicated the presence of sensitive antibiotics. For patients with a preliminary diagnosis of SEA, the empirical scheme used was vancomycin + cefotaxime/ceftriaxone/cefepime/ceftazidime, and antibiotic treatment generally lasted for 4–8 weeks (62). Because of the severe gastrointestinal reaction after vancomycin administration, this patient only empirically selected ceftriaxone. The postoperative drug sensitivity test results suggested that the patient was treated with linezolid and rifampicin for 3 weeks. Re-examination showed that the abscess in the spinal canal had disappeared, and spinal cord compression was relieved. We removed the internal fixations, performed laminectomy, decompressed the vertebral canal, drained the abscess, removed the intervertebral infection and lesion tissues, and implanted vancomycin-loaded calcium sulfate. The main reasons are as follows: (1) internal fixations may be the source of infection, so we removed them, and (2) artificial disc or interbody fusion cage and other implants cannot be planted into the infected intervertebral disc, and the vancomycin-loaded calcium sulfate implantation can achieve long-term interbody fusion, stabilize the spine, and prevent infection.

Because of the rarity of this disease, the acuteness, and the varied range of lesions, there were also deficiencies and irregularities in the diagnosis and treatment: (1) lack of experienced clinicians prevented timely diagnosis and treatment, causing rapid progression of the disease and the spread of abscess; (2) in the first operation, only single-segment laminectomy and abscess drainage were performed, and drainage was insufficient; (3) during the surgery, laminectomy was performed and the pedicle screws were removed, and vancomycin-loaded calcium sulfate was implanted in the intervertebral disc. It remained unclear if instability of the spine in the later period would still require long-term follow-up observation; (4) the abscess was not continuously irrigated with antibiotics after surgery; and (5) the patient was placed on bed after surgery with his head elevated and his foot lowered to utilize gravity for further drainage, but this also caused pus accumulation in the lumbar incision aggravating the infection at that site. Therefore, the case can be used as a reference for early diagnosis, improving the treatment plan, and avoiding future issues.

## 4. Conclusion

Early diagnosis is the key to treating extensive SEA. For patients with back pain who may be suspected with this disease, a comprehensive emergency assessment should be performed immediately. For patients with neck or back pain, spinal cord degeneration, neurological dysfunction, or signs of systemic infection, ESR, CRP, blood culture, and MRI should be performed immediately. Based on neurological tests and medication therapy failure, surgical techniques should be considered if SEA is discovered during imaging. For extensive SEA with clear diagnosis, decompression treatment should be performed at an early stage, when neurological symptoms, spinal cord signal changes, or extensive compression occur. The mode and scope of decompression should follow the principles of fully relieving spinal cord compression and draining purulent secretions with minimal trauma. For patients without neurological symptoms or those with known pathogens who respond to antibiotic treatment, surgical treatment may not be required, but this approach must be undertaken cautiously because neurological dysfunction may be aggravated or become fatal at any time. The treatment duration should be comprehensively evaluated, combining results from the patient's clinical manifestations, laboratory indicators (WBC, ESR, and CRP levels), and imaging examination. Administering broad-spectrum intravenous antibiotics immediately and ongoing medical attention are crucial part for treating extensive SEA. All patients with acute exacerbation should have early surgical intervention for extensive SEA as an adjuvant therapeutic option.

## Data Availability

The original contributions presented in the study are included in the article/Supplementary Material, further inquiries can be directed to the corresponding author/s.
